# Investigating the causal role of the gut microbiota in esophageal cancer and its subtypes: a two-sample Mendelian randomization study

**DOI:** 10.1186/s12885-024-12205-w

**Published:** 2024-04-04

**Authors:** Jia Li, Xuedi Gao, Xiaoming Sun, Hao Li, Jiaheng Wei, Lin Lv, Liangming Zhu

**Affiliations:** 1 Thoracic Surgery Department, Jinan Central Hospital, Shandong University, Jinan, 250000 China; 2 Thoracic Surgery Department, Jinan Mingshui Eye Hospital, Jinan, 250000 China; 3 Thoracic Surgery Department, Jinan Central Hospital, Jinan, 250000 China; 4 Thoracic Surgery Department, Jinan Central Hospital, Shandong First Medical University, Jinan, 250000 China; 5 Thoracic Surgery Department, Weifang Medical University, Weifang, 261000 China

**Keywords:** Mendelian randomization, Esophageal cancer, Esophageal adenocarcinoma, Gut microbiota

## Abstract

**Background:**

Through research on the gut microbiota (GM), increasing evidence has indicated that the GM is associated with esophageal cancer (ESCA). However, the specific cause-and-effect relationship remains unclear. In this study, Mendelian randomization (MR) analysis was applied to investigate the causal relationship between the GM and ESCA, including its subtypes.

**Methods:**

We collected information on 211 GMs and acquired data on ESCA and its subtypes through genome-wide association studies (GWASs). The causal relationship was primarily assessed using the inverse variance weighted (IVW) method. Additionally, we applied the weighted median estimator (WME) method, MR–Egger method, weighted mode, and simple mode to provide further assistance. Subsequent to these analyses, sensitivity analysis was conducted using the MR–Egger intercept test, MR-PRESSO global test, and leave-one-out method.

**Result:**

Following our assessment using five methods and sensitivity analysis, we identified seven GMs with potential causal relationships with ESCA and its subtypes. At the genus level, *Veillonella* and *Coprobacter* were positively correlated with ESCA, whereas *Prevotella9*, *Eubacterium oxidoreducens group*, and *Turicibacter* were negatively correlated with ESCA. In the case of esophageal adenocarcinoma (EAC), *Flavonifractor* exhibited a positive correlation, while *Actinomyces* exhibited a negative correlation.

**Conclusion:**

Our study revealed the potential causal relationship between GM and ESCA and its subtypes, offering novel insights for the advancement of ESCA diagnosis and treatment.

**Supplementary Information:**

The online version contains supplementary material available at 10.1186/s12885-024-12205-w.

## Background

Esophageal cancer (ESCA) is currently one of the most prevalent malignancies globally, ranking 9th in terms of incidence [1], and is associated with a notably high mortality rate, ranking 6th among all tumor types [2]. This cancer can be categorized into two primary pathological subtypes: esophageal squamous cell carcinoma (ESCC) and esophageal adenocarcinoma (EAC) [3]. ESCC is the predominant subtype, accounting for approximately 80% of all ESCA cases, and has the potential to manifest anywhere in the esophagus [2]. Conversely, EAC, which constitutes approximately 20% of ESCA cases, primarily afflicts individuals of Caucasian descent in developed nations, predominantly arising in the distal esophagus or the gastroesophageal junction [4]. Although EAC is not the most common ESCA subtype, its incidence has surged by nearly 60% in recent decades, making it the fastest-growing malignancy [5]. Given the nonspecific early symptoms of ESCA, patients often remain asymptomatic until the cancer has advanced to the middle or late stages, resulting in a generally unfavorable prognosis. Hence, investigating the currently unknown pathogenesis of ESCA has the potential to curtail its incidence and progression, ultimately reducing the mortality rate and improving the overall prognosis for patients with ESCA.

The gut microbiota (GM) represents the largest microecosystem within the human body [6]. An increasing body of evidence underscores the close association between the GM and the onset and progression of various human diseases, including the development of malignancies. Research has revealed that the intestinal microbiota can influence the genesis of ESCAs through diverse mechanisms. For instance, a high-fat diet can induce alterations in the composition of the intestinal microbial flora, resulting in elevated levels of proinflammatory cytokines and immune cells, thereby contributing to tumorigenesis [7]. Similarly, a high-fructose diet can also reshape the intestinal microbial flora, promoting systemic inflammatory responses and metabolic alterations in the host, which are associated with the development of ECA [8]. Furthermore, investigations have revealed notable differences in the composition of the fecal intestinal flora between individuals with ESCA and their healthy counterparts [9]. Despite the identified links between the intestinal flora and ESCA, establishing a definitive causal relationship remains a challenge, primarily due to the presence of confounding factors.

MR experiments, akin to randomized controlled trials, are used to investigate the causal relationships between exposure and outcome factors through the utilization of instrumental variables, which often include single nucleotide polymorphisms (SNPs) [10]. SNPs, by adhering to the principle of random genetic variation allocation, take precedence over disease occurrence, making them effective instrumental variables that circumvent the impact of confounding factors and reverse causality [11]. Compared to randomized controlled trials, MR experiments offer a more accessible means to discern causal links between exposure and outcome factors, and they have been applied in the exploration of causal relationships between the GM and various diseases [12–14]. In the context of this study, we employed a two-sample MR approach to investigate the causal connections between GM and ESCA and to identify protective and risk factors associated with this type of malignancy.

## Materials & methods

### Mendelian randomization study design

**Fig. 1:**
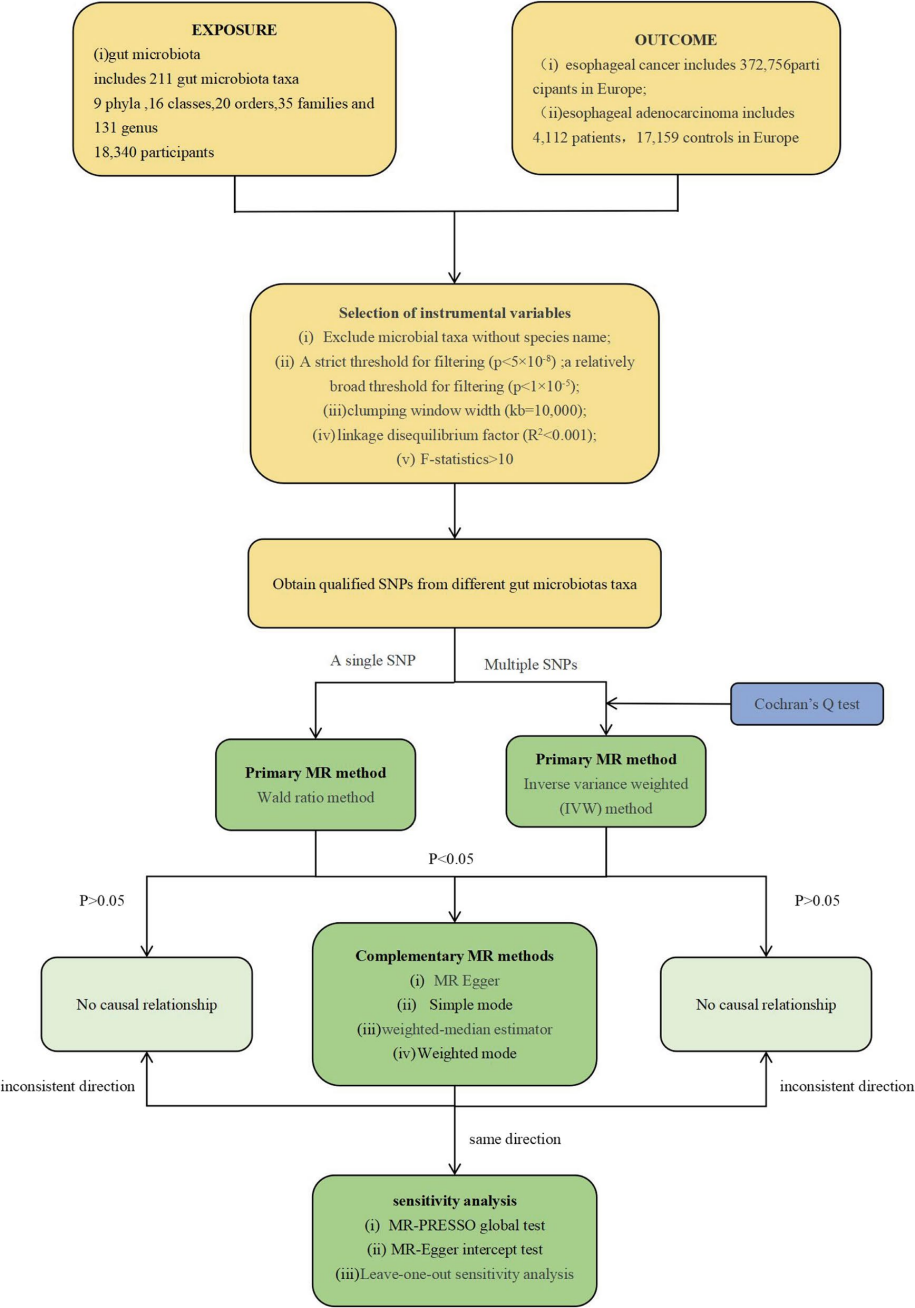
The workflow of the study

The process flow chart of this two-sample MR experiment is depicted in Fig. 1. In our investigation of the causal relationship between the GM and ESCA, we utilized SNPs as instrumental variables (IVs). The selection of these IVs is contingent upon satisfying three critical assumptions [15]. 1. Correlation hypothesis: We chose SNPs associated with ESCA and EAC as instrumental variables, each of which demonstrated a robust correlation with the GM. 2. Exclusion hypothesis: As instrumental variables, the selected SNPs should exhibit no involvement with confounding factors linked to GM, ESCA, or EAC. 3. Exclusionary hypothesis: Under this assumption, instrumental variables solely influence outcome factors through exposure factors, thereby allowing for a more precise two-sample MR analysis in the subsequent stages of the experiment. Our adherence to the STROBE-MR guidelines ensures the methodological rigor of this study [16].

### Data sources

The GM data utilized in this study were sourced from the latest genome-wide association study (GWAS) meta-analysis conducted within the MiBioGen research project [17]. This database encompasses 16S rRNA gene sequencing profiles and genotyping information from a diverse population of 11 adults and adolescents of European and American ancestry, totaling 18,340 participants spanning 24 cohorts. We initially identified 211 GM categories across five taxonomic levels: phylum, class, order, family, and genus. Following the exclusion of 15 unidentified bacterial taxa, we proceeded with a set of 196 taxa for further investigation, encompassing 9 phyla, 16 classes, 20 orders, 32 families, and 119 genera (Supplementary Table S1).

The ESCA and EAC datasets utilized in this study were obtained from the GWAS database. The ESCA dataset includes information from 372,756 participants of European ancestry. In the case of EAC, we specifically selected data from 4112 patients diagnosed with EAC, juxtaposed with 17,159 well-matched controls of European ancestry [18].

Ethical approval was not necessary for the GM data, ESCA data, or EAC data employed in our study. This exemption is attributed to utilizing publicly available GWAS datasets as the primary source of our experimental data. Detailed information regarding the data sources utilized in this study is provided in Table 1.

**Table 1:** Data sources for this study and details of genome-wide association studies

Exposure or outcome	Sample size	Ancestry	Links for data download
Gut microbiome	18,340 participants	Mixed	https://mibiogen.gcc.rug.nl
Esophageal cancer	372,756participants	European	https://gwas.mrcieu.ac.uk/datasets/ieu-b-4960/
Esophageal adenocarcinoma	4112 patients,17,159 controls	European	http://ftp.ebi.ac.uk/pub/databases/gwas/summary_statistics/GCST003001-GCST004000/GCST003739/

### Selection of instrumental variables

In this study, we used SNPs that exhibited a strong association with 196 GM groups as IVs. Initially, we applied a stringent filtering criterion (*p* < 5 × 10^−8^); however, this approach yielded a limited number of IVs. Consequently, we adjusted the filtering threshold to a more permissive level (*p* < 1 × 10^−5^) to secure a more extensive set of IVs for subsequent investigation. Linkage disequilibrium (LD) refers to the phenomenon where genes located at different positions are inherited at a heightened frequency within a biological population [13]. Ensuring the independence of each IV necessitates removing LD among the IVs. We accomplished this by applying a linkage disequilibrium factor (R^2^) threshold of 0.001 and a clumping window width of 10,000 base pairs. Consequently, SNPs that failed to meet these criteria were excluded from the pool of 196 GMs. Additionally, SNPs that were missing data or exhibited palindromic structures were also eliminated, resulting in the retention of the remaining eligible SNPs within each GM group as candidate IVs. Next, we eliminated weak instrumental variables by calculating the proportion of R^2^ and F-statistics. When F-statistics< 10, the SNP was considered a weak instrumental variable and was excluded from subsequent MR studies. The calculation formulas for R^2^ and F-statistics are as follows [19]:

$${R}^2=\frac{2\times EAF\times \left(1- EAF\right)\times bet{a}^2}{\left[2\times EAF\times \left(1- EAF\right)\times bet{a}^2+2\times EAF\times \left(1- EAF\right)\times N\times s{e}^2\right]}$$

$$\textrm{F}=\frac{{\textrm{R}}^2\times \left(\textrm{N}-2\right)}{1-{\textrm{R}}^2}$$

Within the formula, EAF denotes the effect allele frequency, while beta and se correspond to the estimated effect and its standard deviation for each SNP, respectively. The variable N signifies the total number of samples [19].

Subsequently, it is imperative to discern and exclude weak IVs by evaluating the proportion of R^2^ and scrutinizing F-statistics. If the F-statistic fell below the threshold of 10, the SNP was regarded as a weak IV and, consequently, was omitted from subsequent MR analyses.

### Statistical analysis

In this two-sample MR study, we employed a range of methodologies to investigate the causal relationships among ESCA, EAC, and the GM. In the context of MR analysis, a significance level of *P* < 0.05 indicated statistical significance. When a single SNP served as an IV, we utilized the Wald ratio method to establish causality. In cases involving multiple SNPs as IVs, we employed five distinct statistical methods: the inverse variance weighted (IVW) method, weighted median estimator (WME) method, MR–Egger method, weighted mode, and simple mode to ascertain causality. The IVW method was chosen as the primary method because of its robustness in MR analysis. The other four statistical methods functioned as supplementary approaches to validate the results obtained via the IVW method. A causal relationship was deemed plausible only when the findings from all methods aligned with the conclusions of the IVW method. The IVW method utilizes the delta method to combine the effect ratios of each valid IV and amalgamate them via meta-analysis, thus determining the overall impact of IVs on the outcomes [20]. Subsequently, we utilized Cochran’s Q test to assess the heterogeneity among individual SNPs. If significant heterogeneity (*P* < 0.05) was detected, we resorted to the random effects IVW method. Otherwise, the fixed-effects IVW method was applied. The precision of the IVW method relies on the assumption that all SNPs are valid IVs; hence, its accuracy diminishes when invalid IVs are present. To assess causality, we employed the WME, which requires more than 50% valid IVs to yield accurate results [20]. To examine horizontal pleiotropy, we also employed the MR–Egger and MR-Pleiotropy RESidual Sum and Outlier (MR-PRESSO) methods. An outcome with a nonzero cutoff value in the former method indicates the presence of horizontal pleiotropy [21]. The latter method additionally identifies potential outliers among SNPs and contrasts the results before and after their removal [22].

The final step involved a leave-one-out sensitivity analysis, which systematically excludes individual SNPs from the IVs and re-evaluates the stability of the causal relationship, pinpointing SNPs that may exert a significant impact [23]. The presence of SNPs genuinely linked to exposure factors can potentially introduce inaccuracies in the results. Thus, it is imperative to redo the MR analysis after the removal of such SNPs to assess the resilience of the findings.

To provide a more scientifically robust explanation of the causal relationship, we employed the Bonferroni method to establish a threshold for determining multiple comparison significance. This threshold was determined based on the number of distinct classifications within the GM. Specifically, we set the significance threshold at *p* = 0.05/n, where ‘n’ corresponds to the number of unique intestinal flora types. Consequently, the thresholds were as follows: phylum, *p* = 5.56 × 10^−3^ (0.05/9); class, *p* = 3.13 × 10^−3^ (0.05/16); order, *p* = 2.50 × 10^−3^ (0.05/20); family, *p* = 1.56 × 10^−3^ (0.05/32); and genus, *p* = 4.20 × 10^−4^ (0.05/119). A *p* value between 0.05 and the respective significance threshold is considered to indicate a potential causal relationship.

This MR study was conducted using the R program, specifically version 4.2.2. We used the “TwoSampleMR” (version 0.5.7) and “MRPRESSO” (version 1.0) packages as integral components of our investigation.

## Results

### Screening IVs

In our two-sample MR study, we diligently adhered to rigorous screening criteria to exclude ineligible SNPs. This meticulous process resulted in the identification of 2482 eligible SNPs encompassing a diverse spectrum of 196 GM types. Among these, 124 SNPs corresponded to 9 phyla, while 223 SNPs corresponded to 16 classes. Furthermore, 279 SNPs were attributed to 20 orders, 444 SNPs to 32 families, and a significant majority of 1365 SNPs to 119 genera (Supplementary Table S2). Importantly, all F-statistics exceeded a threshold of 10, indicating that all IVs included were free from weak instrument bias.

### MR analysis

#### Impact of intestinal microbiota on ESCA

**Fig. 2:**
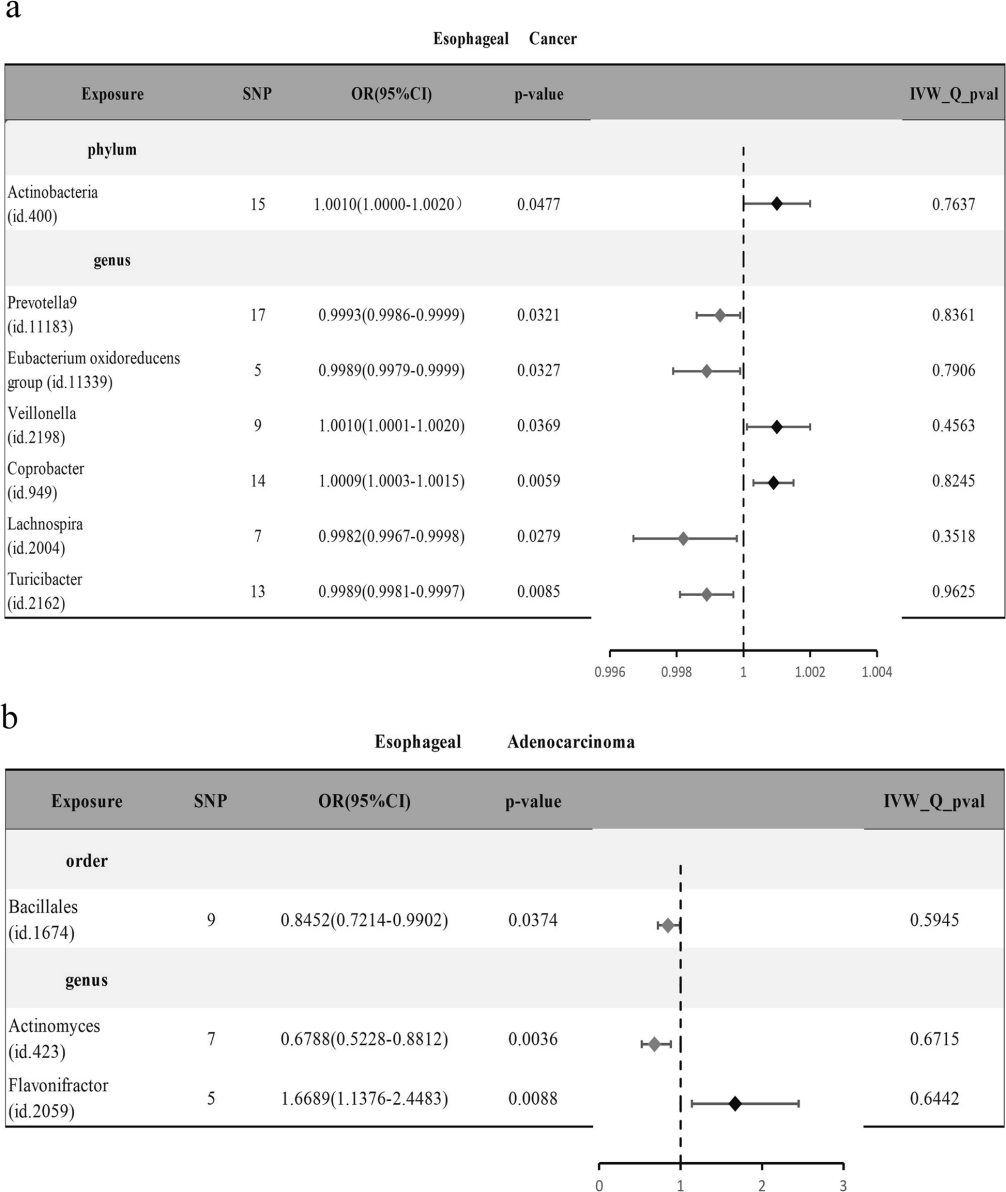
Forest plot of disease-related gut microbiota (GM) identified using the inverse variance weighted (IVW) method: (**a**) esophageal cancer (ESCA) and (**b**) esophageal adenocarcinoma (EAC)

**Fig. 3:**
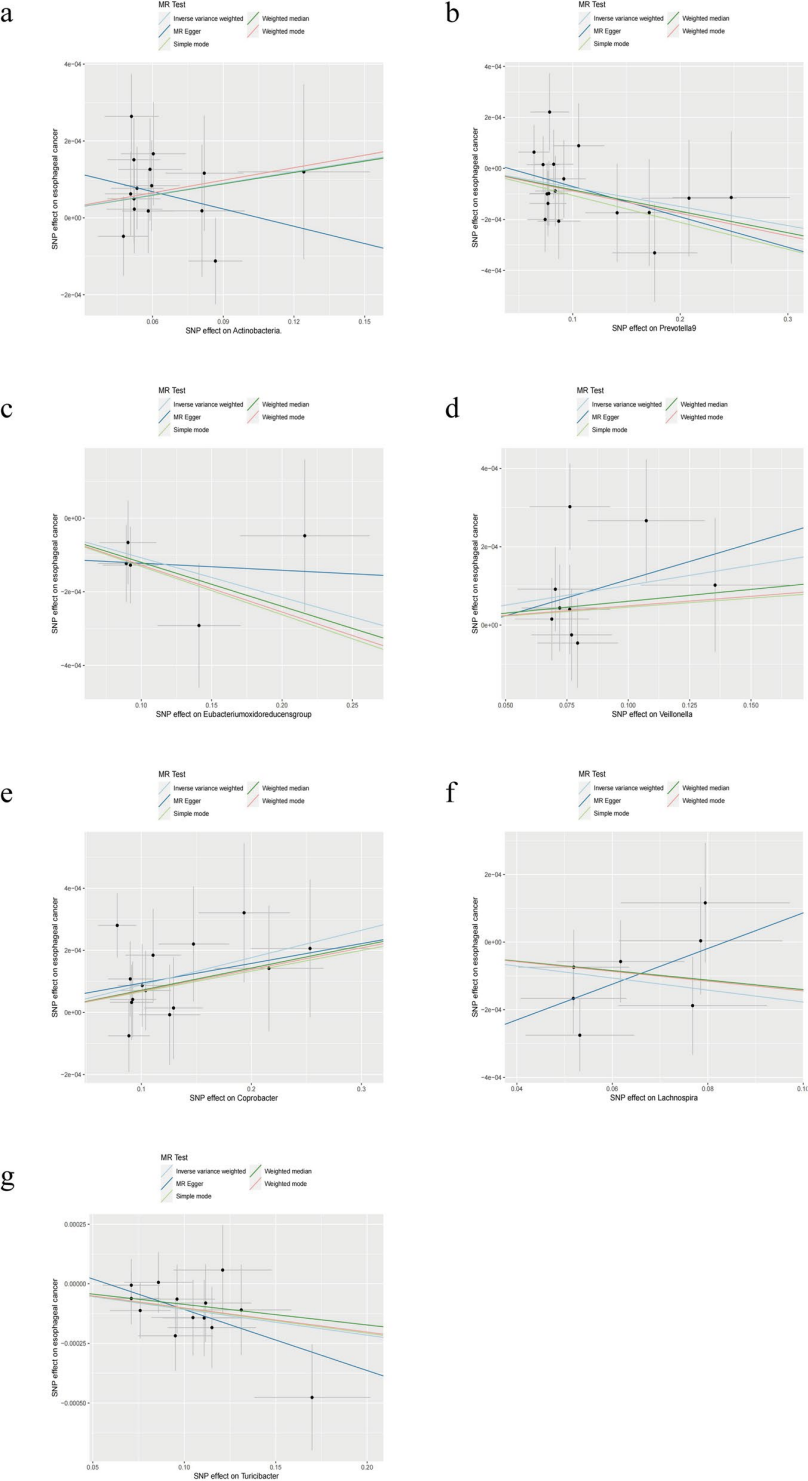
Scatter plot of identifying GMs related to ESCA using IVW, MR–Egger, simple mode, weighted median estimator (WME), and weighted mode methods. (**a**) *Actinobacteria*, (**b**) *Prevotella9*, (**c**) *Eubacterium oxidoreducens group*, (**d**) *Veillonella*, (**e**) *Coprobacter*, (**f**) *Lachnospira*, and (**g**) *Prevotella9*

Initially, we used the IVW method to evaluate the causal relationship between GM and ESCA, and the results are provided in Supplementary Table S3. The IVW analysis revealed that seven specific GMs exhibited a potential causal association with ESCA (*P* < 0.05). These included *Actinobacteria* at the phylum level and *Prevotella9, Eubacterium oxidoreducens group, Veillonella*, *Coprobacter, Lachnospira,* and *Turicibacter* at the genus level (Fig. 2a). Subsequently, we conducted analyses using additional statistical approaches, namely, the WME method, MR–Egger method, weighted mode, and simple mode. Most of these methods yielded conclusions consistent with those of the IVW analysis (Fig. 3b-e, g). However, there were discrepancies in the outcomes of the phylum Actinobacteria (Fig. 3a) and the genus *Lachnospira* (Fig. 3f) between the MR–Egger method and the other four methods. These opposing results prompted us to discount the potential causal relationship between these two GMs and ESCA. Our comprehensive analysis revealed that *Veillonella* (odds ratio [OR]: 1.0010; 95% confidence interval [CI]: 1.0001, 1.0020; *p* = 0.0369) and *Coprobacter* (OR: 1.0009; 95% CI: 1.0003, 1.0015; *p* = 0.0059) were associated with an increased risk of ESCA. In contrast, *Prevotella9* (OR: 0.9993; 95% CI: 0.9986, 0.9999; *p* = 0.0321), *Eubacterium oxidoreducens group* (OR: 0.9989; 95% CI: 0.9979, 0.9999; *p* = 0.0327), and *Turicibacter* (OR: 0.9989; 95% CI: 0.9981, 0.9997; *p* = 0.0085) were associated with a reduced risk of ESCA. Subsequent Cochran’s Q tests for these gut microbiota strains yielded *P* values exceeding 0.05, indicating a lack of heterogeneity (Fig. 2a).

#### Impact of GMs on EAC

**Fig. 4:**
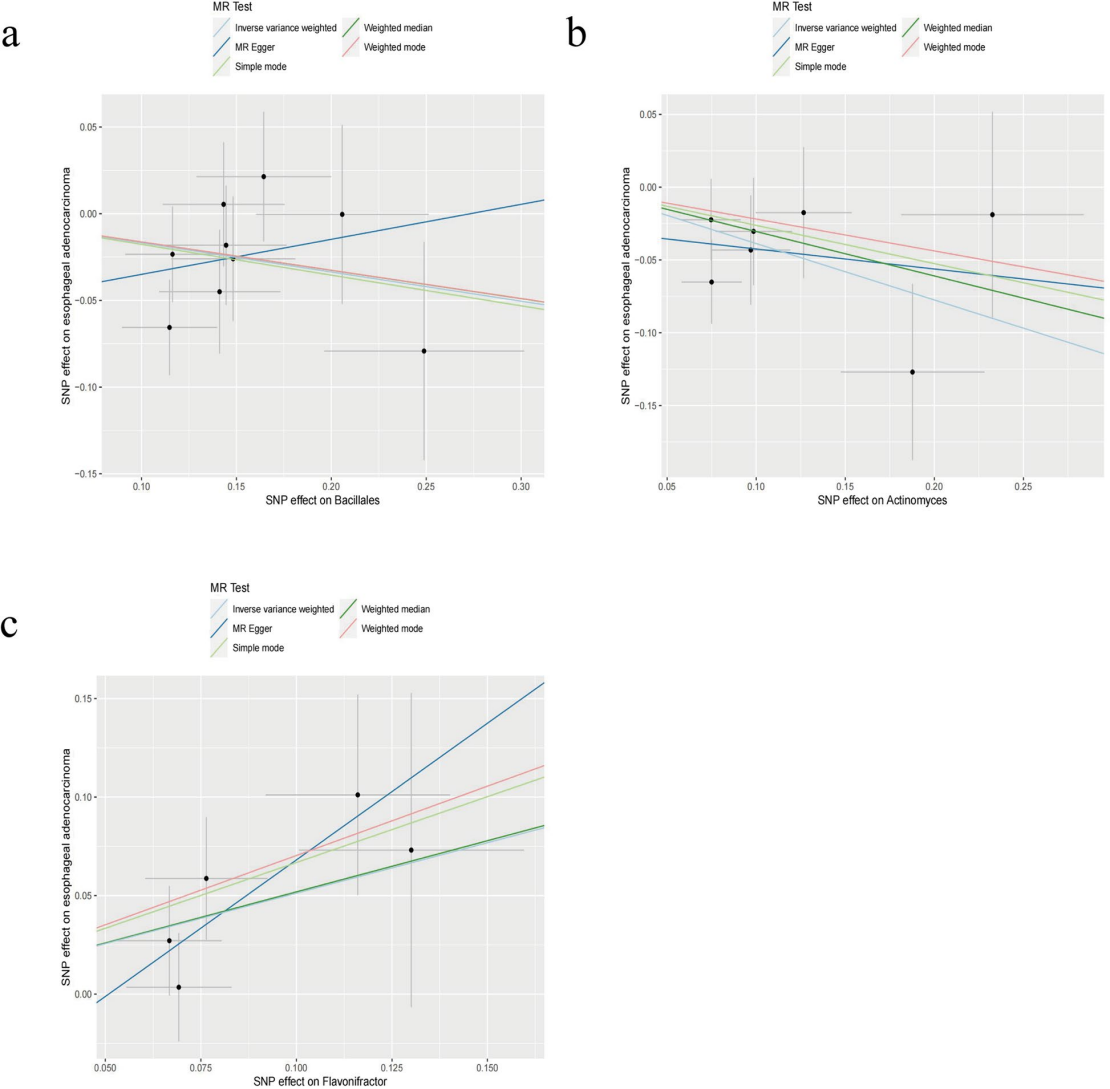
Scatter plot for identifying GMs related to EAC using IVW, MR–Egger, simple mode, WME, and weighted mode methods. (**a**) *Bacillales*, (**b**) *Actinomyces*, (**c**) *Flavonifracto*

We also conducted a similar analysis for EAC. Initially, we employed the IVW method to investigate the potential causal relationship between GMs and EAC, and the results were documented (Supplementary Table S4). Based on the IVW analysis, we derived important insights. Among the 196 GMs under scrutiny, we identified three GMs with potential associations with EAC: *Bacillales* at the order level and *Actinomyces* and *Flavonifractor* at the genus level (Fig. 2b). Subsequently, we employed the WME method, MR–Egger method, and the same weighted mode and simple mode statistical approaches. The outcomes for *Bacillales* from the MR–Egger method contradicted the results of the other four statistical methods (Fig. 4a), leading to its exclusion. However, we retained the results for the remaining two GMs (Fig. 4b, c). In conclusion, we determined that *Flavonifractor* (OR: 1.6689; 95% CI: 1.1376, 2.4483; *p* = 0.0088) was associated with an increased risk of ECA, while *Actinomyces* (OR: 0.6788; 95% CI: 0.5228, 0.8812; *p* = 0.0036) was associated with a reduced risk of ECA. Subsequently, we conducted Cochran’s Q tests for these two intestinal microbiota strains, yielding *p* values exceeding 0.05, which is indicative of the absence of heterogeneity (Fig. 2b).

### Sensitivity analysis

**Fig. 5:**
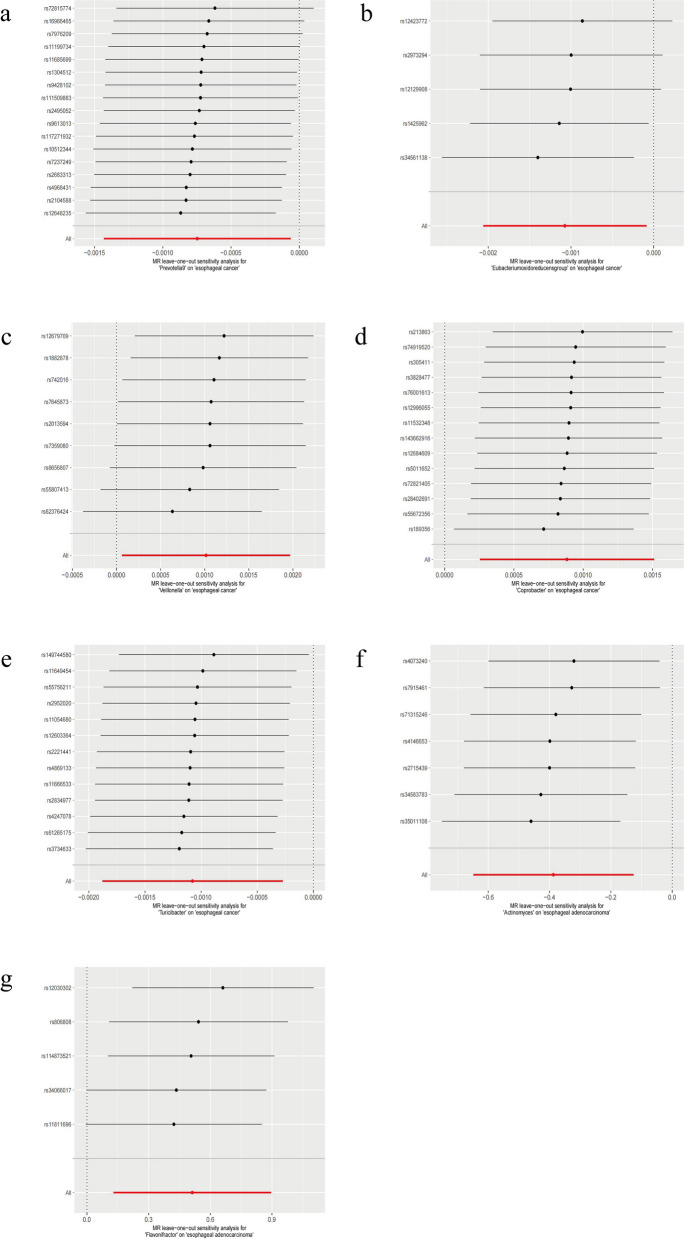
Leave-one-out sensitivity analysis of disease-related GMs. (**a**) *Prevotella9*, (**b**) *Eubacterium oxidoreducens group*, (**c**) *Veillonella*, (**d**) *Coprobacter*, (**d**) *Turicibacter*, (**f**) *Actinomyces*, and (**g**) *Flavonifractor*

Subsequently, we conducted a sensitivity analysis on 7 GMs, which exhibited consistent results across the five statistical methods. Additionally, we performed the MR-PRESSO global test and observed the absence of heterogeneity in the results (PMR-PRESSO > 0.05), as outlined (Tables 2 and 3). Concurrently, the MR–Egger intercept test did not reveal any horizontal pleiotropic effects (PMR-Egger > 0.05) (Tables 2 and 3). Following these assessments, we conducted a leave-one-out sensitivity analysis, confirming the robustness of the MR analysis results. Regardless of which IVs were omitted, the results remained consistent with the original findings (Fig. 5a-g).

**Table 2:** Sensitivity analysis of esophageal cancer (ESCA)-related gut microbiota (GM)

Exposure	MR–Egger intercept test			MR-PRESSO global test	
	Egger_intercept	SE	*p* value	RSS obs	*p* value
genus					
Prevotella9 (id.11183)	4.83E-05	9.13E-05	0.604	11.666	0.86
*Eubacterium oxidoreducens* group (id.11339)	−1.03E-04	1.78E-04	0.604	2.857	0.76
Veillonella (id.2198)	−6.91E-05	2.10E-04	0.751	9.857	0.49
Coprobacter (id.949)	3.06E-05	1.12E-04	0.788	9.402	0.84
Turicibacter (id.2162)	1.48E-04	1.67E-04	0.392	5.773	0.973

**Table 3:** Sensitivity analysis of esophageal adenocarcinoma (EAC)-related GMs

Exposure	MR–Egger intercept test			MR-PRESSO global test	
	Egger_intercept	SE	*p* value	RSS obs	*p* value
genus					
Actinomyces (id.423)	− 2.88E-02	3.89E-02	0.492	5.637	0.709
Flavonifractor (id.2059)	−7.05E-02	7.03E-02	0.390	4.221	0.670

## Discussion

In this two-sample MR study, we systematically explored the causal relationships between 211 GMs and ESCA and its subtypes using a large-scale GWAS database. Our analysis involved stringent screening criteria to exclude weak IVs and mitigate potential confounding factors that could influence the results. Subsequently, we conducted sensitivity analyses aimed at addressing issues related to heterogeneity and horizontal pleiotropy, ultimately ensuring the robustness of the causal inferences we derived. As a result, we identified seven GMs that exhibit potential causal relationships with ESCA and its subtypes.

An increasing body of evidence highlights the existence of causal relationships between GMs and various types of tumors, including those of prostate cancer [24], lung cancer [25], gastric cancer [26], and colorectal cancer [27–30]. Numerous studies on GMs have consistently demonstrated their potential to advance the field of clinical tumor immunotherapy [31, 32]. Moreover, research has established that an imbalance in the intestinal microbial flora represents a major risk factor for ESCA [33]. Nonetheless, since current research on the relationship between intestinal flora and ESCA is mainly observational, the specific types of GMs that exhibit causal associations with ESCA and its subtypes remain unclear. The present study offers a fresh perspective for guiding future treatments of ESCA and its subtypes.

Previous investigations have identified specific intestinal flora capable of stimulating inflammation in the esophageal mucosa by altering their abundance, thereby contributing to degradation [34]. Substantial distinctions in bacterial flora types were observed between patients with ECA and their healthy counterparts. Notably, there was an increase in the abundance of Lactobacilli and *Escherichia coli* [35, 36]. Research indicates variations in both the abundance and functionality of the intestinal flora. In Barrett’s esophagus (BE), microorganisms exhibit tendencies toward repair and replication, whereas in EAC, there is an increase in energy, replication, and signal metabolism potential. There is a decrease in the pathways associated with fatty acid biosynthesis, nitrogen metabolism, and D-alanine metabolism [37].

In our study, we identified a potential causal relationship between the genera *Veillonella* and *Coprobacter* and an elevated risk of ESCA, while the reverse was true for *Prevotella9*, *Eubacterium oxidoreducens group*, and *Turicibacter*. Notably, previous reports have highlighted *Veillonella* as one of the most prevalent normal bacterial species in the esophagus [38], characterized by its gram-negative anaerobic nature. It has been observed that in cases of esophageal anomalies, particularly in the presence of BE, there is a tendency for an increased abundance of gram-negative anaerobes and microaerophiles, including *Veillonella*, which aligns with our study findings. This may be attributed to the transition from gram-positive aerobic bacteria to gram-negative anaerobes, which can stimulate *Veillonella* and other bacterial species due to external environmental factors, potentially leading to pathological changes [39]. Notably, *Veillonella* is recognized as a potentially harmful bacterial species in various other malignancies. For instance, elevated levels of *Veillonella* have been detected in the catheterized urine of bladder cancer patients compared to those in control subjects [40]. Conversely, *Coprobacter* is a bacterial genus known to suppress butyrate production [41]. An elevated abundance of *Coprobacter* can inflict damage upon the intestinal mucosal barrier by producing toxins, hindering bile absorption, competing for nutrients, and releasing antibacterial substances. Consequently, this disruption upsets the equilibrium of intestinal microorganisms [42, 43]. Although there are limited reports of *Coprobacter* in the esophagus, it exhibits significant variations in abundance in other types of tumors. Previous studies have revealed *Coprobacter* to be notably abundant in patients with colon cancer, with a marked prevalence in the proximal colon compared to the distal colon [44]. Furthermore, a substantial surge in *Coprobacter* abundance has been detected in patients with *Neurosyphilis* [45]. Such an increase in *Coprobacter* abundance may lead to the erosion of the gastrointestinal mucosa, facilitating the absorption of deleterious substances and subsequently triggering an inflammatory response. Inflammation is one of the prevalent potential factors contributing to tumor development, consistent with our findings in the context of ESCA. The genus *Prevotella9*, on the other hand, plays a role in the immune response by promoting programmed cell death protein 1 (PD-1) [46], although its performance varies across different tumors. Prior investigations have indicated that patients with unresectable liver cancer tend to exhibit elevated levels of *Prevotella9*, which serves as a risk factor preceding immunotherapy [47]. Conversely, in patients with bladder cancer, the abundance of *Prevotella9* is lower [48]. These disparities may be linked to the intricate variations in immune-inflammatory responses that *Prevotella9* is involved in, contingent on the specific tumor type. Furthermore, *Prevotella9* has emerged as a protective factor in autoimmune conditions such as psoriasis [49], a finding that aligns with our study on ESCA. Nevertheless, the precise underlying mechanisms of these multifaceted immune responses necessitate further exploration. The genus *Eubacterium oxidoreducens group* has been the subject of relatively few prior studies, and the precise mechanisms by which it influences human physiological processes require further investigation. Previous research has indicated that the use of antibiotics can increase the incidence of BE and EAC [50]. Furthermore, long-term antibiotic administration can induce substantial alterations in the composition of the GM, resulting in an increased abundance of Firmicutes and a decrease in the abundance of Bacteroidetes [51]. Notably, the *Eubacterium oxidoreducens group* falls within the Firmicutes category. This finding contrasts with the findings of our earlier study, which identified the *Eubacterium oxidoreducens group* as a protective factor against ESCA. Nonetheless, it is essential to recognize that antibiotics represent a fundamental approach to eradicate *Helicobacter pylori*, a critical factor in ESCA development. The intricate interplay among these factors warrants further exploration. The genus *Turicibacter,* a member of the order *Erysipelotrichales* within the phylum *Firmicutes*, is a gram-positive, obligate anaerobic bacterium [52]. While previous studies have not established a definitive causal relationship between *Turicibacter* and ESCA, some investigations have suggested that *Turicibacter* may serve as a beneficial intestinal bacterium with anti-inflammatory properties [53]. A previous study involving mouse models revealed that riboflavin deficiency led to an increase in *Turicibacter* abundance, subsequently resulting in esophageal epithelial atrophy [54]. Interestingly, in other malignancies, such as liver cancer, *Turicibacter* is considered a protective bacterial species and is negatively correlated with liver cancer [55]. Our study supports the notion that *Turicibacter* may act as a protective bacterial species against ESCA, although the precise underlying mechanisms warrant further investigation.

In our investigation of the ESCA subtype EAC, we identified a potential association between the genus *Flavonifractor* and an increased risk of this cancer, while the genus *Actinomyces* appeared to exhibit protective properties. The genus *Flavonifractor*, a gram-positive anaerobic bacterium belonging to the genus Clostridium, possesses the ability to metabolize catechins [56, 57]. Previous research in mouse models demonstrated that oral administration of *Flavonifractor* drugs effectively reduced Th2 immune responses, thereby suppressing the immune response [58]. However, limited information exists about the role of *Flavonifractor* in esophageal health. In the context of other cancers, such as pancreatic cancer, *Flavonifractor* interacts with blood metabolites, potentially increasing the risk of pancreatic cancer [59]. Additionally, *Flavonifractor* is recognized as an important bacterium in colon cancer [60]. These findings in other cancer types align with our current study on EAC. *Actinomyces*, an anaerobic gram-positive Bacillus commonly found in gastrointestinal and genitourinary flora [61], has been identified as a protective species against BE and EAC in previous studies [35, 62]. These findings align with the outcomes of our current study. However, in the context of other cancers, such as bladder cancer, *Actinomyces* can serve as a pathogenic bacterial species that contributes to tumor initiation and progression [40]. It has been postulated that variations in diet, medications, and other factors among individuals may induce changes in the abundance of the GM through the modulation of metabolites and inflammatory cytokines, such as IL-8, potentially impacting tumor development [6]. The exploration of the underlying mechanisms remains a worthwhile endeavor.

In this study, we employed a two-sample MR approach to investigate the potential causal relationships between GMs and ESCA and its subtypes. We utilized a diverse array of statistical methods to conduct rigorous validations, ultimately identifying seven GMs with potential causal links to ESCA and its subtypes. Furthermore, we conducted sensitivity analyses to ensure the robustness of our findings, offering fresh insights for the diagnosis and treatment of ESCA and its subtypes. Nonetheless, our study had certain limitations and areas that warrant further investigation. Initially, the stringent threshold (*p* < 5 × 10–8) applied to the GWAS resulted in a limited number of IVs. To mitigate this issue, we employed a relatively lenient threshold (*p* < 1 × 10^−5^) for validation. Additionally, the patient data pertaining to ESCA and its subtypes were derived exclusively from European patient samples, which introduced geographical constraints and provided a relatively small sample size. For future research, the utilization of GWAS data encompassing larger sample sizes and diverse ethnic groups is imperative to validate our findings. Furthermore, some of the intestinal bacteria under examination in this study are infrequently documented in previous research or have not been reported within the context of the esophagus. As such, these understudied bacterial species have potential for further exploration.

## Conclusions

The aim of this study was to investigate the causal associations between GMs and ESCA and its subtypes. Our analysis ultimately revealed potential causal relationships between ESCA and its subtypes and 7 GMs: *Veillonella*, *Coprobacter, Prevotella9, Eubacterium oxidoreducens group*, *Turicibacter, Flavonifractor*, and *Actinomyces.* These findings offer novel insights into prospective diagnostic and therapeutic strategies for ESCA and its subtypes.

### Supplementary Information

**Supplementary Material 1.**

**Supplementary Material 2.**

**Supplementary Material 3.**

**Supplementary Material 4.**

## Data Availability

The datasets analyzed during the current study are available in the MiBioGen repository (https://mibiogen.gcc.rug.nl/), the IEU OpenGWAS project (https://gwas.mrcieu.ac.uk/datasets/ieu-b-4960/) and GWAS Catalog (http://ftp.ebi.ac.uk/pub/databases/gwas/summary_statistics/GCST003001-GCST004000/GCST003739/).

## Abbreviations

GM: Gut microbiota
MR: Mendelian randomization
ESCA: Esophageal cancer
GWAS: Genome-wide association studies
IVW: Inverse variance weighted
WME: Weighted median estimator
EAC: Esophageal adenocarcinoma
ESCC: Esophageal squamous cell carcinoma
SNPs: Single nucleotide polymorphisms
IV: Instrumental variable
LD: Linkage disequilibrium
MR-PRESSO: MR-pleiotropy residual sum and outlier
BE: Barrett’s esophagus
